# Comparison of polymerization and structural behavior of microtubules in rat brain and sperm affected by the extremely low-frequency electromagnetic field

**DOI:** 10.1186/s12860-019-0224-1

**Published:** 2019-08-29

**Authors:** Dariush Gholami, Gholamhossein Riazi, Rouhollah Fathi, Mohsen Sharafi, Abdolhossein Shahverdi

**Affiliations:** 1 Institute of Biochemistry and Biophysics (IBB), University of Tehran, Tehran, Iran; 2 Department of Embryology at Reproduction Biomedicine Research Center, Royan Institute for Reproductive Biomedicine, ACER, Tehran, Iran; 3 Department of Poultry Sciences, Faculty of Agriculture, Tarbiat Modares University, Tehran, Iran; 4 Reproductive Epidemiology Research Center, Royan Institute for Reproductive Biomedicine, ACECR, Tehran, Iran

**Keywords:** Brain and sperm, ELEF, Microtubule

## Abstract

**Background:**

Microtubule proteins are able to produce electromagnetic fields and have an important role in memory formation, and learning. Therefore, microtubules have the potential to be affected by exogenous electromagnetic fields. This study aimed to examine the comparison of microtubule polymerization and its structural behavior in brain and sperm affected by 50 Hz extremely low-frequency electromagnetic field (ELEF).

**Results:**

Twenties adult male rats were randomly and equally divided into control and experimental groups, to evaluate the effect of 50 Hz ELEF on the sperm and brain functions. Plus-maze, serum testosterone and corticosterone, and sperm evaluation were performed. Next, the semen and brain samples were obtained, and they were divided into four experimental groups for investigation of microtubule polymerization. There was no significant difference in testosterone and, corticosterone levels, anxiety behaviors, and sperm morphology between control and ELEF-exposure groups. The sperm viability, total and progressive motility were significantly higher in the ELEF-exposed group than that of the control group. The microtubule polymerization in sperm ELEF was significantly higher than in other groups. The secondary and tertiary structures of tubulins were significantly affected in the brain, and sperm ELEF groups.

**Conclusion:**

It seems that the polymerization of microtubules and conformational changes of tubulin dimers are improved by ELEF application.

## Background

Some studies have suggested that electromagnetic fields may affect adversely reproduction and fertilizing potential of spermatozoa [1] as well as biological destructive effects on the mammalian brain [2]. While a number of studies have reported that electromagnetic fields not only did not induce adverse effects on health but also positively induce reproductive systems [3]. Recently, much attention has been paid to the effect of extremely low-frequency electromagnetic field on biological functions of cells and molecules including bone healing [4], the levels of cell calcium [5], and regeneration of injured peripheral nerve using in vitro and in vivo experiments [6, 7]. Low-frequency electromagnetic fields have been reported that influences the actions of intracellular or membrane proteins and induce neural differentiation of bone marrow mesenchymal stem cells without nerve growth factors [7]. Exposure to pulsed electromagnetic fields has resulted in axonal regrowth and acceleration of regenerative neurite outgrowth [8]. Numerous studies have been reported that microtubule polymerization is a key role in axonal growth and nerve regeneration [9].

Microtubules are one of the major components of the cytoskeleton in eukaryotic cells including sperm [10] and nerve cells [11]. They contribute to several processes in cells such as mitosis, secretion of hormones [12], sperm motility [10], chromosomes separation during cell division, and cell movement [13]. Microtubules are 24 nm diameter tubular structures which widely presented in plant and animal cells [14]. The αβ-tubulin dimer assembles dynamically forming these hollow cylindrical structures. This αβ-tubulin family is differentially expressed, highly conserved and post-translational modified [15–17] which are associate in a head-to-tail to form protofilaments and create a dynamic polarity [18]. The tubulin dimer has two GTP binding sites including non-exchangeable and exchangeable GTP site in a protofilament. At the exchangeable site, GTP hydrolysis occurs quicker when the GTP tubulin dimer is bound within the microtubule than when it is released in solution. At the present of sufficient concentration of free GTP tubulin, microtubules will grow and lead to tubulin-GTP cap production on the microtubule. The tubulin-GTP cap is necessary and sufficient to stabilize microtubule. When the growth rate is reduced, tubulin-GTP cap lost because GTP hydrolysis to GDP occurs in the tubulin dimer [19, 20]. In this condition, microtubule undergoes rapid shrinking due to the binding energy of GDP-tubulin is lower than that of GTP-tubulin dimer. This self-polymerization and depolymerization of microtubules are called dynamic instability [21, 22].

By knowing the important role of the microtubule polymerization in the axon regrowth, and nerve regeneration, they have formed one of the most important targets of memory formation [12]. The studies suggested that microtubule polymerization is a very important feature of microtubule protein structures [23]. Neuroplasticity, which is defined as the capacity of neural cells in the formation of new neural connections or modifying the ones has plays a crucial role in memory formation [24]. Therefore, the increase of microtubule polymerization leads to more neural connections and increase memory [23]. The aim of the present study was to investigate the effects of 50 Hz ELEF on microtubule polymerization of the rat sperm and nerve cells.

## Results

### Weight, testosterone, and corticosterone measurement

There was no significant difference in weights between control and ELEF-exposure groups (*p* > 0.05, Table 1). Furthermore, there was no significant difference in testosterone level between control and ELEF-exposure groups (*p* > 0.05, Table 1). There was no significant difference in corticosterone between the control group and ELEF-exposure group (*p* > 0.05, Table 1).

**Table 1:** Body weight, testosterone, corticosterone levels, and elevated plus-maze in the experimental groups

parameters	Groups	
	Control group (*n* = 10)	ELEF-exposed group (*n* = 10)
Body weight (g)	405.16 ± 19.97	400.24 ± 20.21
Testosterone (ng/ml)	5.58 ± 0.3	6.04 ± 0.51
Corticosterone (ng/ml)	21.45 ± 0.42	22.58 ± 0.51
% Open arms entries	34.40 ± 0.64	33.60 ± 1.00
% Open arms time	28.44 ± 0.83	28.76 ± 0.92

Values are expressed as Means ± SD, *significant at *p* < 0.05, independent sample t-test

### Anxiety behaviors in rats

There was no significant difference in the percentage of time in the open arms, and percentage of arms entries between control and ELEF-expose groups, which indicates a stable behavior with unchanged in anxiety (*p* > 0.05, Table 1).

### Sperm quality analysis

Sperm parameters and motion variables in control, and ELEF-exposed groups are presented in Table 2. Total and progressive motility were significantly (*p* < 0.05) higher in the ELEF-exposed group than that of the control group. Moreover, VCL, VSL, VAP, LIN, STR, ALH, and BCF were significantly (*p* < 0.05) increased in ELEF-exposed group in comparison with the control group. However, there was no significant difference in WOB between the control group and ELEF-exposure group (*p* > 0.05). In addition, there was no significant difference in sperm concentration between control and ELEF-exposure groups (*p* > 0.05).

**Table 2:** Sperm quality parameters

Characteristics	Groups	
	Control group *(n =* 10)	ELEF-exposed group (*n* = 10)
Cauda epididymal sperm number (× 10^6^/epididymis)	182.00 ± 0.58	191.00 ± 0.85
Total motility (%)	74.52 ± 0.97^*^	83.42 ± 0.67^*^
Progressive (%)	24.85 ± 1.2^*^	34.08 ± 0.75^*^
Non-progressive (%)	49.67 ± 0.21	49.34 ± 0.48
Motion variables		
VCL (μm/s)	83.57 ± 1.42^*^	127.73 ± 1.38^*^
VSL (μm/s)	42.13 ± 1.1^*^	62.28 ± 1.54^*^
VAP (μm/s)	52.07 ± 1.42^*^	80.21 ± 1.08^*^
LIN (%)	54.94 ± 2.32^*^	71.05 ± 1.42^*^
STR (%)	73.08 ± 1.44^*^	90.50 ± 1.85^*^
WOB (%)	63.24 ± 1.14	61.22 ± 1.07
ALH (μM)	4.17 ± 0.1^*^	9.29 ± 0.14^*^
BCF (Hz)	21.02 ± 1.28^*^	29.27 ± 1.18^*^
Other data		
Viability (%)	70.58 ± 1.25^*^	81.42 ± 1.98^*^
Normal morphology (%)	89.76 ± 1.12	93.25 ± 1.54
Sperm with head defect (%)	4.23 ± 0.46	3.2 ± 0.37
Sperm with tail defect (%)	6.01 ± 0.52^*^	3.55 ± 0.12^*^

Total and progressive motility, and sperm motion variables in the experimental groups. Values are expressed as mean ± SD, * significant at *p* < 0.05, independent sample t-test

### Sperm viability and morphology

Table 2 displays the percentage of the sperm viability and morphology of two experimental groups. The percentage of viability was significantly higher (*p* < 0.05) in the ELEF-exposed group compared with the control group (81.42 ± 4.25 vs. 70.58 ± 5.27%, respectively).

There was no significant difference in the number of sperm with normal morphology in ELEF-exposed group in comparison with the control group (*p* > 0.05). However, the number of sperm with the tail defect was significantly lower (*p* < 0.05) in ELEF-exposed group than that in control group.

### Polymerization assay of microtubule and tubulin purification

**Fig. 1:**
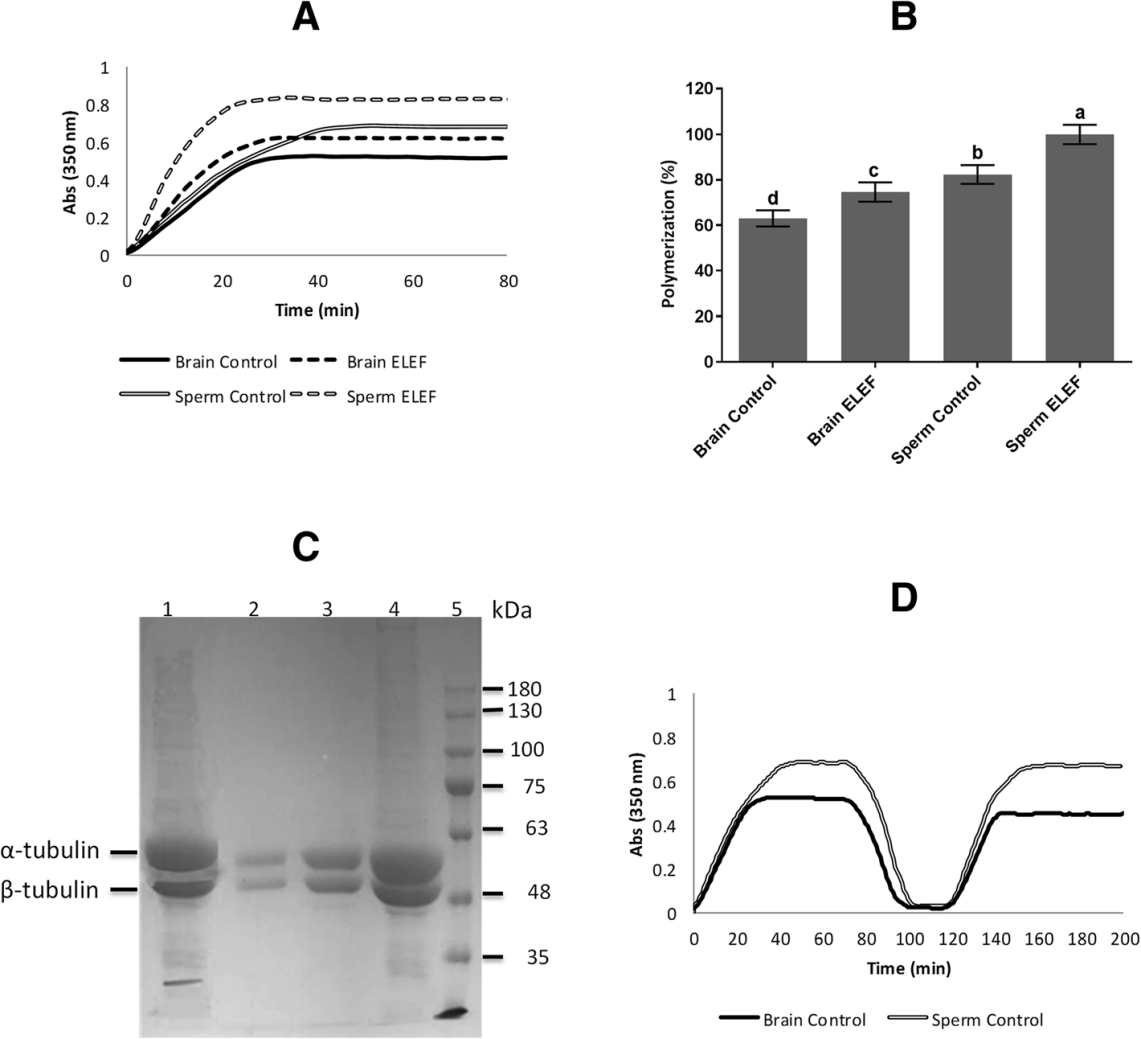
Microtubule polymerization and tubulin purification. **a** The effect of 50 Hz ELEF on microtubule polymerization. **b** Polymerization of microtubule in brain control, brain ELEF, sperm control, and sperm ELEF. **c** Obtained fractions subjected to SDS-PAGE. The lanes are as follows: tubulin dimers prepared by one cycle of polymerization and depolymerization from sperm extracts (lane 1), α/β tubulin dimers purified by phosphocellulose column chromatography from sperm extracts (lane 2), tubulin dimers prepared by one cycle of polymerization and depolymerization from brain extracts (lane 4), α/β tubulin dimers purified by phosphocellulose column chromatography from brain extracts (lane 3), and protein marker (lane 5). **d** Polymerization/depolymerization cycle of microtubules shows the native function of microtubule dynamics. The assigned letters of a, b, c, and d denote significant differences (*P* < 0.05) among the groups. The result is from 3 independent experiments. Values are expressed as mean ± SD, Tukey post hoc test

The polymerization of microtubules in sperm ELEF (100 ± 4.26%) was significantly higher than in sperm control, brain ELEF, and brain control (82.37 ± 4.12, 74.69 ± 4.25, and 63.02 ± 3.56%, respectively, *p* < 0.05). Moreover, this value in sperm control was significantly higher than brain ELEF, and brain control, as well as, this value in brain ELEF was significantly higher than brain control (*p* < 0.05, Fig. 1A, B).

Tubulins achieved by phosphocellulose column chromatography were validated using SDS-PAGE gel electrophoresis to measure the purity of tubulins, and these tubulin proteins were purified with high quality (≥90%, Fig. 1C). The yield of protein concentration extracted from sperm was 0.1 mg/ml and the value extracted from the brain was 2 mg/ml.

### Polymerization/depolymerization cycle of microtubules

Polymerization/depolymerization cycle shows the native function of microtubule dynamics. Therefore, microtubule polymerization was measured at 37 °C for 70 min and thereby microtubules disassembled at 4 °C and repolymerization were monitored at 37 °C. Microtubule aggregation was not detected in the mentioned conditions (Fig. 1D).

### Evaluation of the tubulins polymerization and fluorescence measurements

**Fig. 2:**
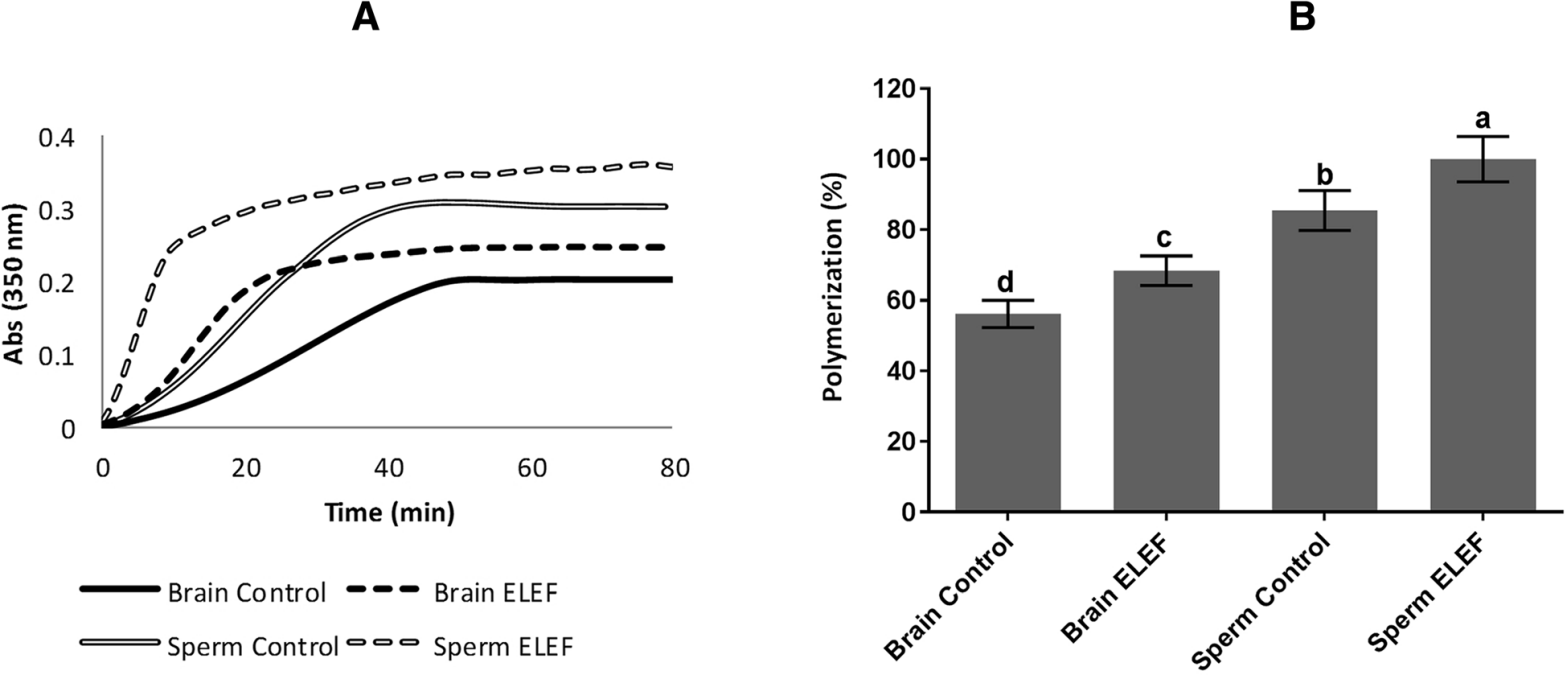
Tubulin polymerization. **a** Graph shows the effect of 50 Hz ELEF on tubulin polymerization. **b** The polymerization of tubulin is significantly different in all experimental groups. The assigned letters of a, b, c, and d indicate significant differences (*P* < 0.05) among the groups. The result is from 3 independent experiments. Values are expressed as mean ± SD, Tukey post hoc test

A significant (*p* < 0.05, Fig. 2A, B) increase in tubulin polymerization of sperm ELEF (100 ± 6.00%) was observed compared to sperm control, brain ELEF, and brain control (85.46 ± 5.64, 68.44 ± 4.18, and 56.18 ± 3.89%, respectively). This value in sperm control was significantly higher than brain ELEF, and brain control. Moreover, tubulin polymerization in brain ELEF was significantly higher than brain control (*p* < 0.05, Fig. 2A, B).

**Fig. 3:**
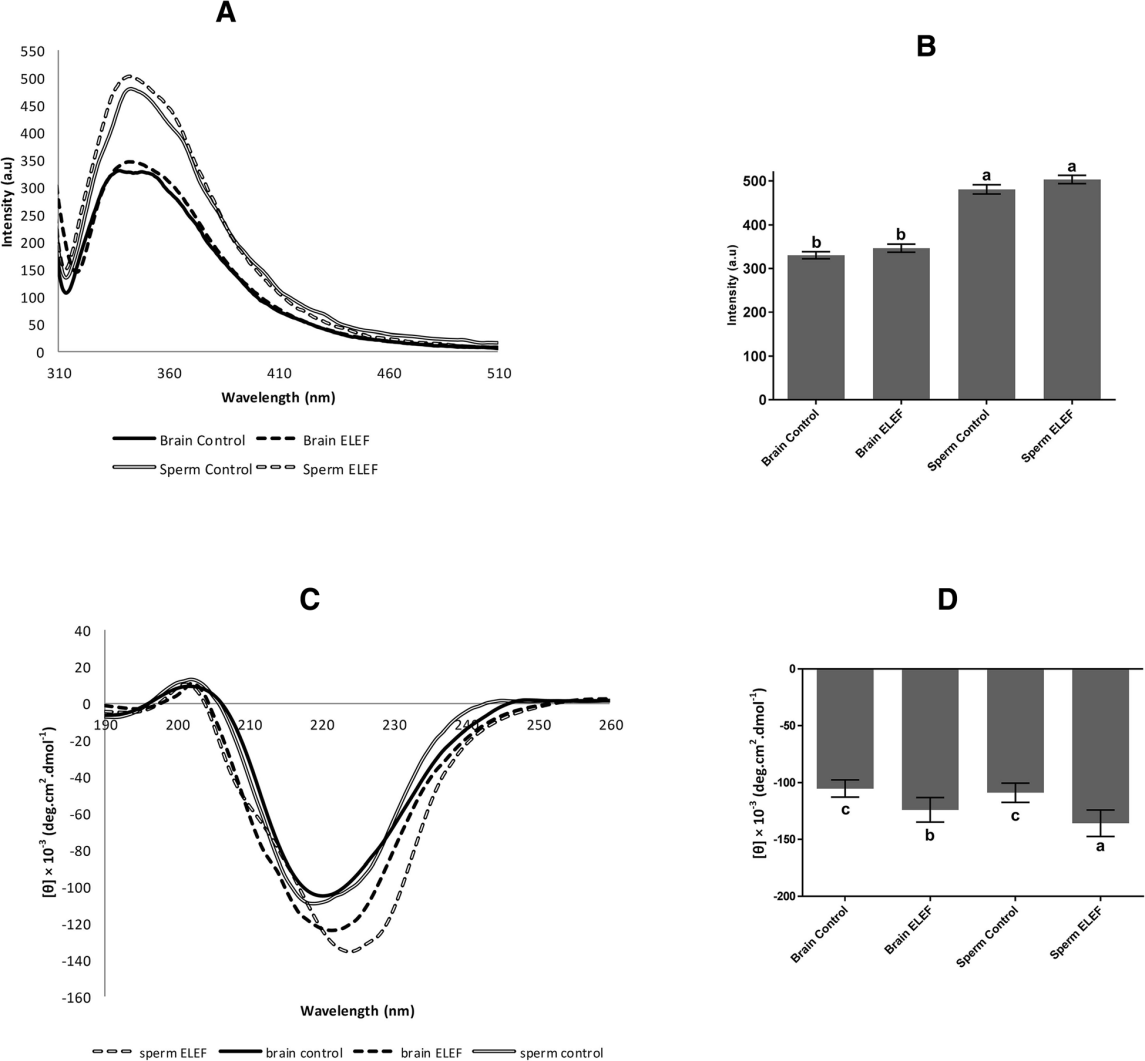
Intrinsic emission and far ultraviolet CD spectra of tubulin dimers. **a** The groups of brain and sperm tubulins show a different intrinsic emission of tubulins. **b** There was no significant differences of emission spectra in the presence of ELEF. **c** The extreme modifications in secondary structure were observed in the presence of ELEF. **d** The changes of ellipticity are presented at 220 nm in sperm, and brain controls, at 221 nm in brain ELEF, and at 224 nm in sperm ELEF. The assigned letters of a, b, c, and d indicate significant differences (*P* < 0.05) among the groups. The result is from 3 independent experiments. Values are expressed as mean ± SD, Tukey post hoc test

Intrinsic emission spectra of tubulin dimers revealed that the emission spectra of sperm control and sperm ELEF were significantly (*p* < 0.05) higher (480.55 ± 10.48, and 503.23 ± 9.56 a.u, respectively) than brain control, and brain ELEF (330.19 ± 8.25, and 346.46 ± 9.27 a.u, respectively). However, there was no significant difference in emission spectra between sperm control vs. sperm ELEF, as well as, between brain control vs. brain ELEF (*p* > 0.05, Fig. 3A, B).

### Tubulin secondary structure by CD spectroscopy

The CD spectrum of the native conformation of sperm ELEF, and brain ELEF tubulins displays a strong negative band of ellipticity values (− 135.75 × 10^− 3^ ± 11.68, and − 123.94 ± 10.86 deg.cm^2^.dmol^− 1^, *p* < 0.05) near 224, and 221 nm, respectively. The weaker negative band of ellipticity values in sperm control, and brain control (− 108.88 × 10–3 ± 8.49, and − 105.16 ± 7.56 deg.cm2.dmol-1, respectively) was observed near 220 nm (*p* < 0.05, Fig. 3C, D).

As shown in Table 3, the secondary structure of tubulins was significantly affected by the 50 Hz ELEF. Analysis of CD spectra demonstrated that the ratio of α-helix content tubulin was significantly (*p* < 0.05) increased in sperm ELEF and brain ELEF in comparison to sperm control and brain control. This value was not significantly different between sperm ELEF and brain ELEF, as well as between sperm control and brain control (*p* > 0.05). The sperm ELEF and brain ELEF significantly had a lower ratio of β-sheet content tubulin compared to sperm control and brain control (*p* < 0.05), while this value was not significantly different between sperm ELEF and brain ELEF, as well as between sperm control and brain control (*p* > 0.05). The ratio of β-turn and random coil values were not significantly different among all of the groups (*p* > 0.05).

**Table 3:** Modifications of the secondary structure of tubulin in four groups

Sample	α-helix (%)	β-sheet (%)	β-turn (%)	Random Coil (%)
Brain Control	30.6 ± 1.24^b^	19.4 ± 1.36^a^	10.7 ± 1.58	39.3 ± 2.58
Brain ELEF	36.12 ± 1.41^a^	14.45 ± 1.24^b^	8.89 ± 0.58	40.54 ± 43
Sperm Control	29.8 ± 2.31^b^	17.7 ± 1.22^a^	11.3 ± 1.65	40.2 ± 2.62
Sperm ELEF	40.14 ± 2.43^a^	12.3 ± 1.14^b^	9.36 ± 1.94	38.2 ± 2.37

The result is from 3 independent experiments. Values are expressed as Means ± SD, *P* < 0.05, Tukey post hoc test

### Tubulin tertiary structure by ANS fluorescence assay

**Fig. 4:**
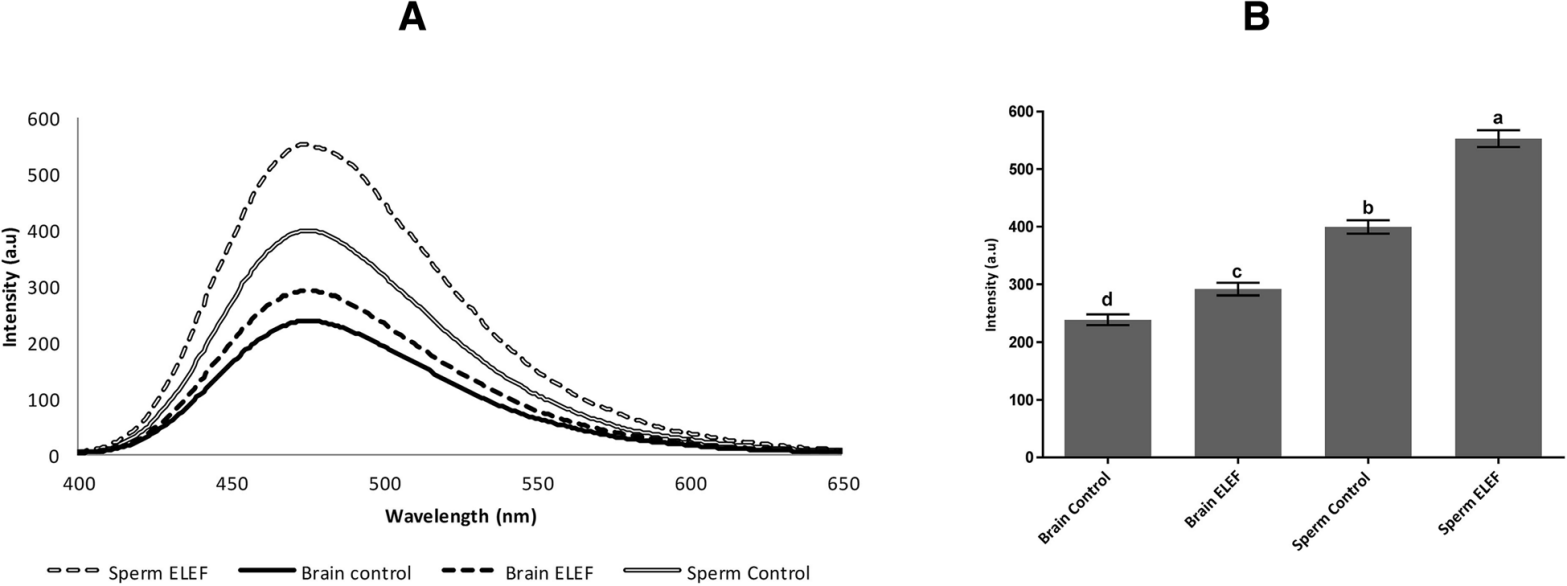
ANS fluorescence assay. **a** and **b** Fluorescence intensity of the tubulin-ANS complex was significantly difference in all experimental groups. The assigned letters of a, b, c, and d denote significant differences (*P* < 0.05) among the groups. The result is from 3 independent experiments. Values are expressed as mean ± SD, Tukey post hoc test

The fluorescence intensity of the tubulin-ANS complex was significantly higher (*p* < 0.05) in sperm control, and sperm ELEF (399.57 ± 11.68, and 552.92 ± 14.56 a.u, respectively) than brain control, and brain ELEF (238.96 ± 9.35, and 291.79 ± 10.72 a.u, respectively). Furthermore, this value was significantly higher in sperm ELEF than sperm control and this value was significantly higher in brain ELEF than brain control (*p* < 0.05, Fig. 4A, B).

## Discussion

In the present study, long- term exposure to 50 Hz ELEF did not change the level of serum testosterone. Consistent with our results, exposure to circularly polarized, sinusoidal, 50 Hz magnetic fields in rats in the periods of 42 days [25], exposure to 50 Hz static magnetic fields on the hypothalamic-pituitary-gonadal axis in the male rat for 40 min/day for 17 days [26], and exposure to magnetic fields 50 Hz, 5 mT intensity in the range of 1–4 weeks’ time exposure [27] did not affect testosterone levels of rats. However, some studies demonstrated that exposure to 128 mT static magnetic fields for 60 min/day for 30 days [28], exposure of 50 Hz sinusoidal magnetic fields for 6 and 12 weeks [29], exposure of radiofrequency electromagnetic field [28], and long term exposure of low frequency electromagnetic fields [30] decreased the levels of serum testosterone in male rats.

We found that exposure to 50 Hz ELEF had no effect on the weight of rats that is consistent with findings reported by Amaral et al. [28], and Bahaodini et al. who stated that long-term exposure to the electromagnetic field emitted from mobile phones had no effect on the weight of rats [30]. Furthermore, studies showed that exposure to the ELF-EMF at 1 Hz and 5 Hz frequencies did not change body weight [31].

The sperm quality analysis showed that sperm motility exposed to square waveform 50 Hz ELEF induced sperm motility. Consistent with our findings, when sperm suspension was exposed to two-phase square wave with 50 Hz frequency, and 5 mT intensity for 2 h of incubation, a significant increase in the motile sperm and in the VAP > 25 μm/s was found [3], also, the VSL and LIN were significantly higher in the similar electromagnetic conditions after 3 h of incubation [3]. However, exposure of sperm suspension to the sinusoidal magnetic field had no effect on the sperm variables [3]. Therefore, it is possible that the 50 Hz ELEF can affect the sperm quality parameters depend on both waveform and amplitude of the applied magnetic field. In addition, some studies demonstrated that exposure to electromagnetic fields had no adverse effects on sperm quality, viability, and morphology [28, 32]. It is probable that ELEF increased sperm viability through reduction of ROS production in sperm cells as previously stated by Gholami et al. [10, 33, 34].

Our findings showed that exposure of 50 Hz ELEF on male rats did not alter serum corticosterone levels. However, some studies were approved of the detrimental effects of electromagnetic exposure on hormones involved in stress responses such as corticosterone. Reports suggested that serum corticosterone concentrations in rats significantly increased during the electromagnetic fields application [35, 36]. Exposure of male rats to 12 Hz electromagnetic waves decreased corticosterone level only day 1 after exposure, while this value did not alter in days 3, 7, 14, and 21 after exposure [36]. Corticosterone is also the primary hormone responsible for the stress response because it is regulated by the HPA axis activated in stressor conditions such as sound, magnetic and electromagnetic fields [37–40]. Increasing corticosterone leads to a neurochemical and neuroanatomical change in the brain, especially in the hippocampus [41]. Hippocampal neurons have the most density of corticosteroid receptors among all neurons in the brain, and these neurons are vulnerable to long-term stress [42]. Also, exposure to long-term stress increases glucocorticoid levels (e.g., corticosterone), and decreases the degeneration threshold of the hippocampal neuron [43, 44].

The elevated plus-maze test is a reliable model for measuring anxiety in laboratory animals that normally uses rodents as a screening test for putative anxiolytic or anxiogenic compounds. The results of the behavioral test in the present study indicate that the time in the open arms and the number of entries to open arms had no difference between groups. These results showed that there was no change in anxiety and depression in the behavior of the 50 Hz ELEF exposure rats. Our results are in line with Sienkiewicz et al. study that showed had no significant effect on the performance of an object recognition task and memory changes in animals exposed with 50 Hz magnetic fields [45]. However, some studies reported adverse effects of electromagnetic fields on anxiety-like behavior of rats. Exposure of male rats to 3 Hz and 60 Hz ELEF/EMF for 2 h/day exposure for 4 days significantly decrease short term memory, and anxiety-like behavior, while this treatment had no effect on both of these values [46]. Vázquez-García et al. reported that exposure to ELEF/EMF of 1 mT intensity during 2 h for 9 days improved the short term memory of male Wistar rats [47].

Several studies have shown the effects of electromagnetic fields on biological systems, and molecular mechanisms. Mobile phone radiofrequency decreased oxygen affinity hemoglobin, and subsequently altered hemoglobin tertiary structure, but the exposed hemoglobin had slightly more secondary structure [48]. In addition, microwaves could change the conformation of proteins that could take the form of a direct interaction of the electromagnetic fields with the proteins or its water of hydration [49]. As microtubules are key proteins that participate in mitosis, secretion of hormones [12], sperm motility [10], cell division, axonal transportation, and neuronal signal transduction [50], it is interesting to examine the effect of electromagnetic fields on microtubule structure and function.

Our results indicated that microtubule and tubulin polymerization in sperm are higher than those in the brain. Although the reason for this is still unknown, it is likely that the important role of microtubule polymerization in sperm motility is a reason for polymerization enhancement [10]. In addition, the multiple isoforms of α- and/or β- tubulin are prevalent among eukaryotes [51], and there are 7α- and 8β- tubulin genes in mice [52]. In the vertebrates a tubulin gene is located at a different genetic locus and encodes for a protein with 400 amino acids in length, and these tubulins are differing in the C-terminal domain which is the most variant and very sensitive to post-translational modifications and these post-translational modifications such as tyrosination, methylation, and acetylation affecting amino acids leads to the production of a variety of tubulin isoforms [52]. The previous studies reported that the expression pattern of a tubulin isoform reflects the role of the microtubule in that specific cell type and pattern of the tubulin expression of other isoforms is differing [51, 52]. For example; Tubα8 is expressed in high levels in the testis and sperm [53]; TubβIVb is predominantly expressed in sperm and testis, while Tubβ1 expressed at high levels in the brain [54]. However, some of the isoforms such as Tuba4a and Tuba8 are expressed in high levels in both sperm and brain because of the important role in the production of high stability microtubules such as axonemes within sperm and axons within neurons [52]. Therefore, it is possible that a difference in microtubule polymerization between sperm and brain is due to the potential tissue-specific expression of different tubulin isoforms. The 50 Hz ELEF increased the polymerization of microtubule proteins due to tubulin structural changes. The data of fluorescence spectroscopy in the present study showed that the structure of tubulin dimers in the presence of 50 Hz ELEF was not unfolded, and because of this, the differences of fluorescence emission were not significant in the presence or absence of ELEF in experimental groups. However, the emission of tryptophan is significantly higher in sperm as compared to the brain because of the more number of tryptophan (17 amino acids) in α/β tubulin dimers in sperm than that in the brain (8 amino acids) [55, 56].

The fluorescence probe ANS is a beneficial compound for the surface hydrophobicity study of proteins. ANS binds to the exposed hydrophobic packet of proteins resulting in large fluorescence enhancement [10, 48]. The results showed that tubulin-ANS complex emission in α/β tubulin dimers in sperm was higher than that in the brain, while the total number of aromatic amino acids including phenylalanine, tyrosine, and tryptophan in α/β tubulin dimers in brain is equal to that in sperm. This can return to the position of aromatic amino acids in the protein structure, and since the formation of a hydrophobic packet is depends on the type of amino acid and its position on the protein structure, differences in the position of amino acids in sperm and brain tubulin dimers affected the tubulin-ANS complex emission.

Furthermore, we previously reported that ELEF induces the conformational modifications in the secondary and tertiary structures of tubulin due to changes in the tubulin oscillation pattern. This phenomenon leads to alter bending angle of α and β tubulin dimers which are necessary for tubulin-tubulin assembly for microtubule establishment [10]. In addition, it is possible that ELEF may affect electron clouds around tubulin dimers by changing the unpaired electrons of hydrophobic regions, and therefore, enhanced microtubule stability [10, 50].

## Conclusions

In this study, the polymerization and structural behavior of tubulins and microtubules in sperm cells and brain of rats in the presence of 50 Hz ELEF was studied. The polymerization of microtubule and tubulin were significantly higher in sperm cells as compared to brain cells. Furthermore, the activity of sperm and brain microtubules was strongly increased by applying 50 Hz ELEF because of the enhancement of microtubule polymerization and conformational changes of tubulin dimers.

## Methods

### Chemicals

All chemicals used in the present study were purchased from Sigma (St. Louis, MO, USA) unless otherwise stated.

### Animals

All experimental animals were obtained from the fully ventilated animal house of the Institute of Biochemistry and Biophysics (IBB), University of Tehran, Iran. The experimental protocols were conducted in accordance with the Animal Care and Use Protocol, University of Tehran, Tehran, Iran. Twenty adult male Wistar rats (250–300 g) were used in this study. They had free access to food and water and were maintained on a 12 h light/dark cycle at 20–22 °C. All rats were adapted to laboratory condition for 7 days before beginning the study and handled in order to reduce the overall stress 10 min before experiments. Animals were randomly divided into two groups including the control group without any treatment, and the experimental group exposed to 50 Hz ELEF as ELEF-exposed group, and they weighed daily in the experimental period. At the end of the experiment, the rats were anesthetized with chloroform and separated by guillotine and their remaining carcasses were transferred to the IBB animal care center.

### Electromagnetic exposure conditions

The ELEF-exposed group was exposed to ELEF with a square waveform of 5 mT intensity and 50 Hz frequency and the maximum induced electric field was 4.1 mV/m, according to Faraday’s law, 24 h/day for 85 days using the Helmholtz coil (The radius of each coil 35 mm, 70 mm high, copper wire, 1000 turns/m, the diameter of the wire in each coil 1.7 mm, self-inductance L = 3 mH, ohmic resistance = 3 Ω) as electromagnetic field generator. The temperature was monitored using a chromel-alumel thermocouple, during the electromagnetic treatment and ∆θ was < 0.01 °C.

### Testosterone measurement

The rats were anesthetized by ether at the end of the exposure period, and 1 ml of blood samples were taken from the heart via cardiac puncture. The blood samples were stored in tubes without anticoagulants, allowed to clot, and centrifuged at 2180 g for 15 min. The serum was stored at − 20 °C until use. Serum testosterone level was assessed by the Testosterone ELISA Kit (cat # ab108666) according to the manufacturer’s instructions. The signal intensity of the samples was read using an ELISA reader (Spectra Max M2e, Molecular Devices, USA) at 450 nm.

### Measurement of corticosterone

Rats were anesthetized with chloroform, and the blood samples were taken directly from the heart of rats. The blood serum was separated by centrifugation at 1600 g for 10 min and corticosterone measured by the Corticosterone ELISA Kit (Cat # KA0468) according to the manufacturer’s instructions. The absorbance of the samples was read on an ELISA reader at a wavelength of 450 nm.

### Anxiety behaviors in rats

The apparatus for EPM test is made of Plexiglas consisted of two open arms (50 × 10 cm) with 1 cm high edge and two closed arms of the maze which were enclosed by 40 cm high walls. The connection of the four arms forms a central square (10 × 10 cm), and the maze was elevated 50 cm from the ground. The experiment took place in an isolated room without disturbing sound in a comfortable environment. The indirect light was located at 1.30 m above the maze, and the activity and place of animals were monitored by a camera located above the apparatus. Two experimental groups were placed in the center of the plus-maze facing an opened arm, and animals moved freely in different parts of the maze within 5 min. The number of times that the animal entered into the open arms was recorded.

### Sperm preparation and quality analysis

For the collection of sperm samples, the cauda epididymis was excised and immersed in 5 ml pre-warmed HBSS to release all sperms, and incubated at 37 °C and gently shacked the Petri dish for 10 min.

The number of sperm was determined with hemocytometer using microscopic examination. Briefly, ten microliters of sperm suspension was loaded into a hemocytometer chamber and the sperm was allowed to settle by keeping the hemocytometer in a humid place for 10 min. The number of sperm in the squares of the hemocytometer was counted under a light microscope at 10× magnification. The sperm concentration was referred to as sperm per epididymis.

The CASA system (Version 5.1; Microptic, Barcelona, Spain) was used to sperm quality analysis. Six microliters of sperm suspension was placed in a Makler chamber, and the total motility (%), progressive motility (%), non-progressive motility (%), VCL (μm/sec), VSL (μm/sec), VAP (μm/sec), LIN (%), STR (%), WOB (%), ALH (μm), and BCF (Hz) were evaluated by CASA.

### Sperm viability and morphology

For assessment of the normal and abnormal morphology, 20 μl of each sample was placed on a slide and air-dried. The smears were then stained by Papanicolaou staining. Two hundred sperms were counted for each sample and percentages of abnormalities were determined by light microscopy at 100× magnification.

For determination of viable sperm, fractions of sperm suspension of each sample was mixed with an equal volume of 0.05% eosin-nigrosin staining, and smear on a microscope slide. The slides were visualized with light microscopy and about 200 vital (unstained) and dead (stained) sperm were counted [57].

### Sperm and brain microtubule extraction

For sperm microtubule extraction, the tubulin dimers were purified from sperm control (sperm that prepared from control group including 10 animals), and sperm ELEF (sperm that prepared from ELEF-exposed group including 10 animals). The sperm (1.5 g) was homogenized by hand homogenizer in 7 ml of PEM buffer containing (100 mM PIPES pH 6.9, 2 mM MgSO_4_, 1 mM EGTA) on ice, and the homogenate was centrifuged at 3000 g for 30 min at 4 °C. The supernatant was incubated and polymerized by adding 0.5 mM Mg^2+^GTP at 37 °C for 45 min. Next, 33% glycerol (V/V) was supplemented to microtubules and centrifuged at 120000 g for 45 min at 25 °C. The pellet was suspended in PEM buffer and microtubules were depolymerized at 4 °C and centrifuged at 85000 g for 45 min at 4 °C. The supernatant containing the tubulin and MAPs was collected and stored at − 70 °C for polymerization experiments. In order to the separation of tubulins from MAPs, an anion exchange chromatography in a phosphocellulose column (25 ml bed volume) was used for further purification [10]. The column was washed three times with PEM buffer, and about 1 mg/ml of protein was loaded onto the column with a constant flow of 1 ml/min. The protein concentration was measured using Bradford reagent (Bio-Rad, Hercules, USA) and BSA was used to protein quantification as standard.

A similar method was used to extract the microtubule from brain control (brain that prepared from control group including 10 animals), and brain ELEF (brain that prepared from ELEF-exposed group including 10 animals).

The purity of extracted tubulins from sperm and brain were over 90% considered by SDS-PAGE electrophoresis. All samples stored at − 70 °C for the next experiments.

### Turbidity assessments

Turbidity measurement was performed with 0.2 ml of tubulin (0.1 mg/ml) in PEM buffer in the presence of 1 mM Mg^2+^ GTP. Next, the polymerization was evaluated at 37 °C by a Cary 100 Bio UV/visible spectrophotometer equipped with a thermometer (Varian, Australia). The process was detected at 350 nm absorbance [10]. In addition, the UV/visible spectrophotometer was set at 4 °C and monitored at 37 °C for the investigation of tubulin depolymerization/repolymerization.

### Fluorescence spectroscopy

Intrinsic fluorescence of extracted tubulins from brain control, brain ELEF, sperm control, and sperm ELEF were monitored at 4 °C. The emission of tubulins was monitored at 295 nm excitation in a range of 300 to 500 nm [58]. Moreover, for ANS fluorometry, solutions containing 2.5 μM tubulins were incubated with 50 μM ANS at 4 °C for 10 min. The emission of sample solutions was monitored at 380 nm excitation in a range of 400 to 650 nm [59]. The mentioned experiments were carried out with Cary Eclipse fluorescence spectrophotometer.

### Circular dichroism spectroscopy

The secondary and tertiary structure of α/β tubulins purified from the brain control, brain ELEF, sperm control, and sperm ELEF were analyzed by a CD spectrophotometer, model 215 (Aviv Biomedical, USA). For the investigation of the secondary structure, the far-ultraviolet CD spectra were monitored in a range of 190 to 260 nm [60]. In addition, for the determination of tertiary structure, the near-ultraviolet CD spectra were detected from 260 to 320 nm [61]. The spectrum of a blank containing buffer and the other reagents was subtracted from all spectra. Deconvolutions of CD spectra and data analysis were performed by CDSD and CDNN programs.

### Statistical analysis

The analysis of the obtained data was performed by the SPSS software version 16 (SPSS Inc. Chicago, ILL). For the evaluation of the testosterone, body weight, sperm parameters quality, and plus-maze test, the data were subjected to a Kolmogorov-Smirnov test of normality and analyzed by independent sample t-test.

One-way ANOVA analysis was used to the measurement of the data in microtubule and tubulin investigation. Afterward, a Tukey post hoc analysis was done for comparing between means. *P*-values less than 0.05 were considered significant.

## Data Availability

All datasets on which the conclusion of the paper relies are available to readers.

## Abbreviations

ALH: amplitude of lateral head displacement
ANS: 8-Anilinonaphthalene-1-sulfonic acid
BCF: beat-cross frequency
BSA: bovine serum albumin
CASA: computer-assisted sperm analysis
CD: circular dichroism
EGTA: ethylene glycol tetra-acetic acid.
ELEF: extremely low frequency electromagnetic field
ELF-EMF: Extremely low frequency- electric and magnetic fields
ELISA: enzyme-linked immunosorbent assay
EPM: elevated plus-maze
GTP: Guanosine-5′-triphosphate;
HBSS: Hanks Balanced Salt Solution buffer
HPA: hypothalamus-pituitary-adrenal
LIN: linearity
MAPs: microtubule-associated proteins
SDS-PAGE: sodium dodecyl sulfate-polyacrylamide gel electrophoresis
STR: straightness
VAP: average path velocity
VCL: curvilinear velocity
VSL: straight linear velocity
WOB: wobble
